# Models of support for disclosure of HIV status to HIV‐infected children and adolescents in resource‐limited settings

**DOI:** 10.1002/jia2.25157

**Published:** 2018-07-04

**Authors:** Elise Arrivé, Samuel Ayaya, Mary‐Ann Davies, Cleophas Chimbetete, Andrew Edmonds, Patricia Lelo, Siew Moy Fong, Kamarul Azahar Razali, Kouadio Kouakou, Stephany N Duda, Valériane Leroy, Rachel C Vreeman, the IeDEA Pediatric Working Group

**Affiliations:** ^1^ ISPED Centre INSERM U1219‐ Epidémiologie‐Biostatistique Université de Bordeaux Bordeaux France; ^2^ INSERM U1219 Centre Inserm Epidémiologie et Biostatistique Université de Bordeaux Bordeaux France; ^3^ School of Medicine College of Health Science Moi University Eldoret Kenya; ^4^ University of Cape Town Cape Town South Africa; ^5^ Newlands Clinic Harare Zimbabwe; ^6^ Department of Epidemiology Gillings School of Global Public Health The University of North Carolina at Chapel Hill Chapel Hill NC USA; ^7^ School of Public Health The University of Kinshasa Kinshasa Congo; ^8^ Hospital Likas Kota Kinabalu Malaysia; ^9^ Hospital Kuala Lumpur Kuala Lumpur Malaysia; ^10^ CIRBA Abidjan Cote D'Ivoire; ^11^ Vanderbilt University School of Medicine Nashville TN USA; ^12^ Inserm U1027 Université de Toulouse 3 Toulouse France; ^13^ Indiana University School of Medicine Indianapolis IN USA; ^14^ Academic Model Providing Access to Healthcare (AMPATH) Eldoret Kenya

**Keywords:** disclosure, adolescents, low‐ and middle‐income countries, vertical HIV infection, counselling, site assessment

## Abstract

**Introduction:**

Disclosure of HIV status to HIV‐infected children and adolescents is a major care challenge. We describe current site characteristics related to disclosure of HIV status in resource‐limited paediatric HIV care settings within the International Epidemiology Databases to Evaluate AIDS (IeDEA) consortium.

**Methods:**

An online site assessment survey was conducted across the paediatric HIV care sites within six global regions of IeDEA. A standardized questionnaire was administered to the sites through the REDCap platform.

**Results:**

From June 2014 to March 2015, all 180 sites of the IeDEA consortium in 31 countries completed the online survey: 57% were urban, 43% were health centres and 86% were integrated clinics (serving both adults and children). Almost all the sites (98%) reported offering disclosure counselling services. Disclosure counselling was most often provided by counsellors (87% of sites), but also by nurses (77%), physicians (74%), social workers (68%), or other clinicians (65%). It was offered to both caregivers and children in 92% of 177 sites with disclosure counselling. Disclosure resources and procedures varied across geographical regions. Most sites in each region reported performing staff members' training on disclosure (72% to 96% of sites per region), routinely collecting HIV disclosure status (50% to 91%) and involving caregivers in the disclosure process (71% to 100%). A disclosure protocol was available in 14% to 71% of sites. Among the 143 sites (79%) routinely collecting disclosure status process, the main collection method was by asking the caregiver or child (85%) about the child's knowledge of his/her HIV status. Frequency of disclosure status assessment was every three months in 63% of the sites, and 71% stored disclosure status data electronically.

**Conclusion:**

The majority of the sites reported offering disclosure counselling services, but educational and social support resources and capacities for data collection varied across regions. Paediatric HIV care sites worldwide still need specific staff members' training on disclosure, development and implementation of guidelines for HIV disclosure, and standardized data collection on this key issue to ensure the long‐term health and wellbeing of HIV‐infected youth.

## 1 Introduction

Disclosure of HIV serostatus to perinatally HIV‐infected children and adolescents is an important part of paediatric HIV care. HIV is a highly stigmatized disease requiring lifelong treatment, but also prevention counselling to avoid onward sexual and mother‐to‐child transmission. In addition, disclosure often involves explaining that the infection was acquired from a biological parent. It is therefore a complex process requiring a comprehensive assessment and appropriate involvement of children's cognitive, psychological, familial and social environments. Partial disclosure (without mentioning HIV/AIDS) can be started early (six to eight years) but should be completed by a full disclosure (with explicit information about his/her HIV infection, HIV disease, AIDS, care and HIV transmission modes) by the age of 12 years the latest, as recommended by the World Health Organization (WHO) 1. The potential emotional and social impact of HIV disclosure could aggravate behavioural disorders, familial conflicts and social stigma 2, 3. However, disclosure is important to improve adherence to antiretroviral therapy (ART) 4 and retention in care 5, as well as to promote safer sexual practices to prevent secondary transmission. Existing data suggest that the HIV disclosure process often occurs late and in the adolescent period 6, 7, 8, 9, 10, 11, 12, 13, 14. Also, the prevalence of children and adolescents aware of their HIV status varies by setting and by the age of the patients, and has been reported to be from 13 to 60% in low‐ and middle‐income countries (LMICs) 6, 7, 8, 9, 10, 11, 12, 13, 14.

With improved effectiveness of ART programmes for perinatally infected children, the numbers of adolescents who have grown up with HIV infection are increasing. Within this population, disclosure represents a new challenge for families, medical staff members and the adolescents themselves 15. To face this challenge and support families, healthcare workers may need disclosure‐specific guidance, skills and programmes. The WHO recommends that caregivers should be oriented to engage at appropriate ages with disclosure process which should be monitored and supported until full disclosure is achieved 16. However, the disclosure process and associated services have not been well characterized across LMICs. The resources available for training providers in these countries about paediatric HIV disclosure are largely based on the Western disclosure model and experience 17. While some models guiding disclosure have been implemented within research settings in LMICs 18, 19, 20, the depth of implementation of such models for HIV status disclosure in resource‐limited, routine clinical care is unclear. Documenting existing strategies for the HIV disclosure process in these settings would help to characterize the current limits and strengths to HIV disclosure process, so that these data could be used to develop supportive interventions. Our objective was to describe current site characteristics related to the process of disclosure of HIV status within paediatric HIV care in LMICs.

## 2 Methods

An online site assessment survey was conducted across HIV care and treatment clinics caring for children within the International Epidemiology Databases to Evaluate AIDS (IeDEA) cohort consortium. IeDEA is an international research consortium established in 2005 by the National Institute of Allergy and Infectious Diseases (http://www.iedea.org/home/who-we-are). IeDEA collects HIV/AIDS data from seven international regional data centres, including four in Africa, and one each in the Asia‐Pacific region, the Caribbean, Central and South America region (CCASA), and North America 21, 22. In the present study, focusing on LMICs, the North America region was not included. All IeDEA‐participating sites and their corresponding central regional data centres have ethics approvals for the collection of patient‐level data within the IeDEA data mergers and for routine surveys of site‐level characteristics. Because the present survey did not include patient‐level data from respondents or patients, and was a part of the routine site‐level surveys, a separate ethics review was not required.

A standardized questionnaire was constructed and administered through the web‐based REDCap platform (http://project-redcap.org/), as part of a site‐level survey about adherence and support services for HIV‐infected children and adolescents. The questionnaire was available in English and French.

All sites caring for children and still engaged in IeDEA consortium at the time of the survey were included in the study. Coordinators at each site were asked to determine the most appropriate person (one by site) to complete the survey as the person‐in‐charge of paediatric HIV services at each clinical site.

Questions were asked about the site (setting, level, public, size…), whether the staff members of the site had received training on counselling related to disclosure of HIV status to children, the site offered disclosure counselling services, the site had a protocol for disclosure of HIV status to children, the site had disclosure status collected routinely and if so, the frequency and method of collection. We also asked the site representative to estimate the percentage of children at the site knowing that they are HIV‐infected by the time they reach 14 years of age. No specific guidance was provided on what was considered for the survey to be disclosure counselling, training, protocol or data collection.

Frequencies and percentages were produced for categorical variables, with medians and interquartile ranges (IQRs) calculated for continuous variables.

We compared the report of disclosure services according to the reported percentage of children knowing their HIV status (<80% or ≥80%) using Chi square or Fisher exact tests. The threshold for significance was 0.05. The analyses were conducted with SAS software version 9.3.

## 3 Results

From June 2014 to March 2015, all 180 sites caring for children and still engaged in IeDEA consortium at the time of the survey in 31 countries of the six regions completed the online questionnaire (response rate of 100%); the majority (53%) were from Southern Africa, and were integrated clinics serving both adults and children (86%) (Table 1). In Asia, CCASA, Central, and West Africa, most of the sites were urban (94% to 100%) and represented regional, provincial, or university hospitals (94% to 100%). The Southern Africa sites were mainly health centres (60%), and in East Africa, they were district hospitals (50%). In both of these regions, the sites were mostly rural (57%). Survey respondents were mainly paediatricians (62%), clinical officers (14%), medical officers (6%), nurses (5%), non‐paediatrician physicians (4%).

**Table 1:** Site characteristics of the paediatric IeDEA consortium by region (N = 180 sites)

	Asia (N = 16; 9%)	Latin America (N = 7; 4)	Central Africa (N = 18; 10%)	East Africa (N = 33; 18%)	Southern Africa (N = 95; 53%)	West Africa (N = 11; 6%)	Total
Site characteristics	N (%)	N (%)	N (%)	N (%)	N (%)	N (%)	N (%)
Location							
Urban	15 (94)	7 (100)	17 (94)	12 (36)	41 (43)	11 (100)	103 (57)
Rural	1 (6)		1 (6)	21 (64)	54 (57)		77 (43)
Clinic type							
Paediatric	13 (81)		1 (6)	1 (3)	3 (3)	7 (64)	25 (14)
Both adult and children	3 (19)	7 (100)	17 (94)	32 (97)	92 (97)	4 (36)	155 (86)
Level							
Health centre	2 (12)		1 (6)	13 (39)	56 (60)	1 (19)	73 (41)
District hospital				16 (49)	24 (25)	2 (18)	42 (23)
Regional, provincial, or university hospital	13 (82)	7 (100)	17 (94)	4 (12)	7 (7)	8 (73)	56 (31)
Unknown	1 (6)				8 (8)		9 (5)
Respondent's role							
Clinician	1 (6)			4 (12)	78 (82)	1 (9)	84 (47)
Principal investigator	12 (75)	4 (57)	1 (5)	2 (6)	9 (9)	4 (36)	32 (18)
Head clinician/clinical officer	2 (12)	2 (29)	8 (45)	22 (67)	2 (2)	3 (27)	39 (22)
Head nurse			6 (33)	1 (3)	1 (1)		8 (4)
Site manager			3 (17)	3 (9)	4 (4)	1 (9)	11 (6)
Other	1 (6)	1 (14)		1 (3)	1 (1)	2 (18)	6 (3)

The clinics each reported caring for a median of 162 children (IQR: 81 to 351) during the previous 12 months.

Almost all the sites (98%) reported offering disclosure counselling services (Table 2). Disclosure resources and procedures varied across regions: staff members' training on disclosure was done in 72% to 96% of sites in each region, with lower frequencies in Central and East Africa. A disclosure protocol was available in 14% (Southern Africa) to 71% (CCASA) of sites within the region, and HIV disclosure status was collected routinely in 50% (Central Africa) to 91% (West Africa) of sites within the region. Eighty‐three percent had at least three of the services or resources described above available. The main collection method of disclosure status was through asking the caregiver and child (85%) about the child's knowledge of his/her HIV status. Disclosure status was collected every three months in 63% of these sites and every month in 26%. Two‐thirds of the sites collecting disclosure status (71%) stored this data electronically; 88% of the sites with electronic disclosure data were from Southern and East Africa.

**Table 2:** Site characteristics related to disclosure of HIV status to HIV‐infected children and adolescents in the paediatric IeDEA consortium by region (N = 180 sites)

	Asia (N = 16)	Latin America (N = 7)	Central Africa (N = 18)	East Africa (N = 33)	Southern Africa (N = 95)	West Africa (N = 11)	Total
Site characteristics	N (%)	N (%)	N (%)	N (%)	N (%)	N (%)	N (%)
Disclosure counselling	16 (100)	6 (86)	18 (100)	31 (94)	95 (100)	11 (100)	177 (98)
Staff members' training on disclosure	15 (94)	6 (86)	13 (72)	24 (73)	91 (96)	10 (91)	159 (88)
Disclosure protocol	9 (56)	5 (71)	10 (56)	11 (33)	13 (14)	5 (45)	53 (29)
Years since a protocol has been initiated; median (IQR)	4.6 (3.5 to 6.0)	11.3 (10.3 to 12.2)	5.7 (2.7 to 5.7)	1.3 (1.2 to 2.9)	4.6 (2.5 to 6.7)	6.1 (5.6 to 7.6)	4.6 (1.4 to 7.1)
Collection of disclosure status	13 (81)	4 (57)	9 (50)	23 (70)	84 (88)	10 (91)	143 (79)
Disclosure status collection method							
Interview with caregiver	2 (15)	3 (75)	2 (22)	6 (26)	2 (2)	1 (10)	16 (11)
Interview with child				5 (22)			5 (4)
Interview with caregiver and child	11 (85)	1 (25)	7 (78)	12 (52)	82 (98)	9 (90)	122 (85)
Frequency							
Every month	1 (8)	2 (50)	6 (67)	22 (96)	2 (2.5)	4 (45)	37 (26)
Every three months	4 (31)	1 (25)	2 (22)		79 (94)	3 (33)	89 (63)
Every six months	4 (31)				1 (1)		5 (3)
Once a year	2 (15)	1 (25)	1 (11)				4 (3)
Other	2 (15)			1 (4)	2 (2.5)	2 (22)	7 (5)
Disclosure status data stored electronically	5 (38)	2 (50)	2 (22)	11 (48)	79 (94)	3 (30)	102 (71)
Estimated percentage of children knowing their HIV status by the age of 14 Median (Interquartile Range)	83 (75 to 95)	95 (20 to 100)	87 (75 to 95)	70 (30 to 90)	80 (80 to 80)	50 (10 to 70)	80 (75 to 80)

Among the 53 sites (29%) with a formal disclosure protocol, 32 (60%) had designed it locally and 21 (40%) borrowed/adapted from various external sources, including four from WHO guidelines, five from local national ART treatment guidelines, and 12 from independent sources (Table 2). For those sites with a disclosure protocol, the protocol was initiated a median of 4.6 years prior to the survey (IQR: 1.4 to 7.1), with a site from CCASA having a protocol for 16.7 years.

Disclosure counselling was most often provided by counsellors (88% of sites), but also by nurses (78%), physicians (76%), social workers (69%), or other clinicians (66%) (Table 3). Counselling was offered to both caregivers and children in 92% of the 177 sites providing disclosure counselling. Almost all the sites (95%) involved caregivers in the disclosure process, but nine did not (Table 3). Counsellors were the healthcare workers most often involved in the disclosure process (74% to 100% of regional sites outside of Asia) except in Asia, where physicians participated more. Nurses were also frequently involved in East and Southern Africa (82% and 94% of regional sites, respectively).

**Table 3:** Persons involved in services to support disclosure of HIV status to HIV‐infected children and adolescents in the paediatric IeDEA consortium by region (N** = 180 sites)**

	Asia (N** = **16)	Latin America (N** = **7)	Central Africa (N** = **18)	East Africa (N** = **33)	Southern Africa (N** = **95)	West Africa (N** = **11)	Total
	N (%)	N (%)	N (%)	N (%)	N (%)	N (%)	N (%)
Persons delivering disclosure counselling							
Physicians	16 (100)	3 (50)	9 (50)	9 (29)	87 (92)	10 (91)	134 (76)
Nurses	7 (44)	4 (67)	10 (56)	25 (81)	89 (94)	3 (27)	138 (78)
Others clinicians	3 (19)	1 (17)		23 (74)	87 (92)	3 (27)	117 (66)
Counsellors	7 (44)	6 (100)	16 (89)	23 (74)	94 (99)	10 (91)	156 (88)
Social workers	4 (25)	5 (83)	12 (67)	15 (48)	80 (84)	6 (55)	122 (69)
Peers			1 (6)	1 (3)			2 (1)
Other				1 (3)	4 (4)		5 (3)
Persons benefiting from disclosure counselling							
Caregivers	2 (12)		1 (6)	7 (23)		1 (9)	11 (6)
Children	1 (6)			2 (6)			3 (2
Both caregivers and children	13 (82)	6 (100)	17 (94)	22 (71)	95 (100)	10 (91)	163 (92)
Persons participating in the disclosure process							
Caregivers	16 (100)	5 (71)	16 (89)	32 (97)	94 (99)	8 (73)	171 (95)
Physicians	14 (87)	6 (86)	12 (67)	12 (36)	87 (92)	8 (73)	139 (77)
Nurses	10 (62)	2 (29)	12 (67)	27 (82)	89 (94)	1 (9)	141 (78)
Other clinicians	3 (19)		2 (11)	21 (64)	86 (91)	3 (28)	115 (64)
Counsellors	7 (44)	6 (86)	17 (94)	23 (70)	94 (99)	10 (91)	157 (87)
Social workers	6 (37)	5 (71)	12 (67)	15 (45)	81 (85)	4 (36)	123 (68)
Peers			2 (11)	1 (3)			3 (2)
Others					4 (4)		4 (2)
Person collecting disclosure status							
Physicians	11 (69)	4 (57)	4 (22)	12 (36)	82 (86)	7 (64)	120 (67)
Nurses	9 (56)	1 (14)	3 (17)	14 (42)	81 (85)		108 (60)
Others clinicians	2 (12)	1 (14)		19 (58)	78 (82)	2 (18)	102 (57)
Counsellors	5 (31)	2 (29)	7 (39)	11 (33)	82 (86)	7 (64)	114 (63)
Social workers	4 (25)	1 (14)	8 (44)	7 (21)	79 (83)	4 (36)	103 (57)
Peers				1 (3)			1 (1)
Other	1 (6)			1 (3)			2 (1)

The median site percentage of HIV‐infected adolescents estimated by the site representatives as knowing their serostatus by age 14 was 80% (IQR: 75% to 80%) (Table 2). Sites with disclosure percentage of 80% or more reported higher frequency of paediatrician as a respondent, disclosure counselling, staff members' training on disclosure and collection of disclosure status but fewer frequency of disclosure protocols (Table 4).

**Table 4:** Site characteristics related to disclosure of HIV status to HIV‐infected children and adolescents in the paediatric IeDEA consortium according to the estimated percentage of children knowing their HIV status by the age of 14 Median (N** = 180 sites)**

Estimated percentage of children knowing their HIV status by the age of 14	<80%	≥80%	*p* value
	N (%)	N (%)	
Paediatrician responding	16 (33)	95 (72)	<10^−4^ a
Disclosure counselling	46 (96)	131 (99)	0174^b^
Staff members' training on disclosure	34 (71)	125 (95)	<10^−4^ a
Disclosure protocol	25 (52)	28 (21)	<10^−4^ a
Collection of disclosure status	34 (71)	125 (95)	<10^−4^ a

^a^Chi squared test; ^b^Fisher exact test.

## 4 Discussion

To the best of our knowledge, this study is the first characterizing HIV disclosure practices among a large sample of paediatric HIV clinical sites (N = 180) in LMICs. Most sites reported offering disclosure counselling services. Current guidelines do not characterize well recommended services associated to disclosure process 1. We have therefore focused on those which were frequently reported in the literature as being important, such as counselling, training, protocol and collection of disclosure process 23, 24. These services, except for the protocol, were associated here with a percentage of children knowing their HIV status by 14 years of age estimated by site representatives of 80% or more. Our study showed that counsellors were frequently the primary individuals involved in the disclosure process in our sites. However, in the literature describing interventions to support disclosure, there is no specific mention of counsellors having a role in this process 25, as usually “healthcare workers” or “providers” are cited as being involved with disclosure. Counsellors and social workers may have been combined into the designation of “provider,” and play key roles in the delivery of care. Further study to document their knowledge level, and attitudes, and practices relevant to disclosure, as well as their working conditions regarding disclosure (e.g. time and effort allocated, training received), could guide future interventions to strengthen counsellors’ skills to enhance the quality of disclosure.

Only one‐third of the sites had a protocol for the disclosure process, reflecting the absence of specific disclosure guidelines and standardized procedures at the site level 26. Collection of disclosure status was not systematic among many of the participating sites, despite these data being useful for routine paediatric HIV follow‐up, particularly for targeting adherence support and transitions from paediatric care to adult care. In addition, these data would help clinic staff members to avoid inadvertent disclosure of a child's status. Electronic capture of disclosure status was less common and is a particular barrier for conducting research on disclosure in these settings. Systematic collection of disclosure status would facilitate inclusion of this factor in retrospective or prospective studies related to clinical, immunological, virological and psychosocial outcomes.

Across the sites, the median percent of children knowing their HIV status by 14 years of age estimated by the site representatives was high at 80%. This is in contrast with the literature, which has frequently reported lower proportions of children/adolescents aware of their HIV status, often less than 50% 13, and at an older age at disclosure. There are several significant limitations to the disclosure estimate reported here. First, this survey was not intended to measure disclosure prevalence directly with families or patients. The primary objective was to assess site services with the survey. The disclosure percentage was estimated roughly by the person in charge of paediatric HIV care, without any requirement for patient data or underlying numbers to calculate this estimate. They may have reported higher rates of disclosure in the context of discussing paediatric services and guidelines that likely push for child HIV disclosure 1, 16.

Another limitation of this survey was the reliance on self‐reported practices, which were not cross‐checked with the site and which may vary from the actual delivery of disclosure services. What we report here may be therefore the optimal higher end of service delivery and what actually happens might be somewhat or even much worse. Finally, the sites participating in this survey were predominantly in urban settings, except in Southern Africa and the survey may characterize resources reflecting the highest levels of HIV care and treatment for children in these countries. However, the majority of these sites were routine care clinics that provided standard and representative HIV care, often in partnership with the Ministries of Health. A few sites were more focused on research. The HIV care provided by these sites was generally considered representative of the care available in each location 27.

## 5 Conclusion

While the majority of IeDEA paediatric sites reported offering disclosure counselling services, educational and social support resources and data collection capacity around disclosure varied by region. Disclosure counselling was mainly delivered by counsellors rather than formal medical staff members, training support was inconsistent, and moderate to low proportions of disclosure protocol use was reported in all the regions. To design appropriate interventions for paediatric HIV disclosure, more information about counsellors’ knowledge, attitudes, practices and working conditions is needed, as is more information about the disclosure training content and categories of healthcare workers who received specific disclosure‐related training. Paediatric HIV care sites worldwide still need specific support on HIV disclosure to ensure the long‐term health and wellbeing of HIV‐infected youth.

## Competing interests

The authors declare not conflict of interest.

## Authors’ contributions

EA contributed to the writing of the protocol, the questionnaire, and the manuscript, and analysed the data. MAD, CC, PL, SMF, KAR, KK and AE contributed to data collection, and reviewed and edited the manuscript. SND contributed to data collection and preparation, and review of the manuscript. VL contributed to the writing of the questionnaire and data collection and reviewed and edited the manuscript. SA and RCV contributed to the writing of the protocol and the questionnaire, data collection and reviewed and edited the manuscript.
